# *Corybaspapillatus* (Orchidaceae), a new orchid species from peninsular Thailand

**DOI:** 10.3897/phytokeys.183.71167

**Published:** 2021-10-08

**Authors:** Janejaree Inuthai, Sahut Chantanaorrapint, Manop Poopath, Naiyana Tetsana, Wittawat Kiewbang, Somran Suddee

**Affiliations:** 1 Department of Biotechnology, Faculty of Science and Technology, Thammasat University, Lampang Campus, Hang Chat, Lampang, 52190, Thailand Thammasat University Lampang Thailand; 2 PSU Herbarium, Division of Biological Science, Faculty of Science, Prince of Songkla University, Hat Yai, Songkhla, 90110, Thailand Prince of Songkla University Songkhla Thailand; 3 Forest Herbarium, Department of National Parks, Wildlife and Plant Conservation, 61 Phahonyothin Road, Chatuchak, Bangkok 10900, Thailand Department of National Parks, Wildlife and Plant Conservation Bangkok Thailand; 4 Forest Economics Bureau, Royal Forest Department, Bangkok 10900, Thailand Forest Economics Bureau, Royal Forest Department Bangkok Thailand

**Keywords:** Helmet orchid, Khao Luang National Park, montane forest, Thai-Malay Peninsula

## Abstract

A new species, *Corybaspapillatus*, is described and illustrated from peninsular Thailand. The new species is easily recognized through a combination of the following characters: the purplish flower, the rounded apex of the dorsal sepal, the outer surface of dorsal sepal covered with irregular papillae in the upper half, the lateral sepals adnate laterally at the base to the connate petals, the V-shaped throat, the labellum bearing short hairs, dentate to erose labellum margins, and well-developed conical spurs. A key to the species of *Corybas* in Thailand is presented.

## Introduction

*Corybas* Salisb. is a genus of terrestrial orchids comprising about 120 species, and is widely distributed from India, South China, peninsular Thailand, the Malesian region, to New Zealand and the Western Pacific Islands (Dransfield et al. 1986; Pridgeon et al. 2001; Pedersen 2011; Tandang et al. 2020). The members of the genus are easily recognized by being small in size with a single cordate leaf and 1–2 underground tubers, dorsal sepal and labellum which together form a tube with expanded mouth, lateral sepals and petals often antenna-like, and labellum usually bearing two spurs (Dransfield et al. 1986). Only two *Corybas* species are currently known from Thailand, namely *Corybasecarinatus* Anker & Seidenf. (Anker and Seidenfaden 2001; Pedersen 2011), and *C.geminigibbus* J.J. Sm. (Chantanaorrapint and Chantanaorrapint 2016).

During a recent visit to Khao Luang Mountain, Khao Luang National Park, by staff of BKF herbarium, an interesting taxon of the genus *Corybas* was collected with a unique combination of characters that did not match any of the known species. It is therefore described here as a species new to science.

## Materials and methods

This study is based on material collected during July 2018 from Khao Luang National Park, Nakhon Si Thammarat province, southern Thailand. Specimens were preserved in alcohol (70% ethanol) and deposited in BKF herbarium. Morphological characters were studied using a stereo microscope Olympus SZX7 and the distinctive characters of the species were illustrated with the aid of an Olympus drawing tube. Measurements were taken from spirit material. The specimen details were compared in detail with original drawings and descriptions given in the protologues of *Corybas* species in the Malaysian region (e.g. Dransfield et al. 1986; Anker and Seidenfaden 2001; Tandang et al. 2020).

## Taxonomy

### Corybas papillatus

Taxon classificationPlantaeAsparagalesOrchidaceae

Inuthai, Chantanaorr. & Suddee sp. nov.

EF98AB20-DF14-505B-9220-50381FC8848E

urn:lsid:ipni.org:names:77220208-1

Figs 1 , 2

#### Diagnosis.

Similar to *Corybasvillosus* J. Dransf. & Gord. Sm., but differs in the absence of dorsal sepal keel and the lateral sepals adnate laterally at the base to the connate petals.

#### Type.

Thailand. Nakhon Si Thammarat province, Khao Luang National Park, near summit of Khao Luang Mt., 08°29'36.8"N, 099°43'38.9"E, ca. 1,700 m alt., 4 July 2018, *M. Poopath, N. Tetsana, W. Kiewbang, C. Hemrat & S. Jirakorn 2201* (holotype BKF!, spirit material).

Small terrestrial herb with underground tubers. ***Tubers*** globose or ovoid, fleshy, 3–4 mm diam. ***Stem*** erect, whitish-green, 5–12 mm long, 1–1.5 mm diam., with a basal sheath; stolon whitish, hairy, up to 2 cm long, 1–1.2 mm diam. ***Foliage leaf*** sessile, cordate, long acuminate at apex, glabrous, 6–10 mm long, 6–8 mm wide at the widest point, flat, only slightly undulate along margin, pale green with paler veins, the veins scarcely conspicuous. ***Inflorescence*** one-flowered, terminal; bract pale green, lanceolate-triangular, long acuminate, 5–6 mm long, recurved. ***Flower*** dark purple. ***Dorsal sepal*** purplish, erect below, then strongly curved above, hooded and clasping labellum throughout its length, spathulate, ca. 15 mm long, 8–10 mm wide, rounded at apex, apical margins denticulate, abaxial (dorsal) surface bearing irregular papillae in the upper half. ***Lateral sepals*** greenish-white, linear-triangular to antenna-like, ca. 25 mm long, laterally adnate to the petals in the basal ca. 1 mm. ***Petals*** greenish-white, connate at the base for ca. 1 mm, free above, similar to lateral sepals but shorter, ca. 22 mm long. ***Labellum*** differentiated into 2 parts, erect in basal half and strongly reflexed above, ca. 17 mm long in total length; the basal half with margins strongly inrolled throughout, and forming a tube with the dorsal sepal, ca. 7 mm long; the upper half reflexed and expanded, orbicular, ca. 10 mm diam., margin erose, reddish-purple excepted for a white zone just below the margins; mouth of throat very deeply V-shaped; adaxial surface bearing short hairs; spurs 2, divergent, conical, ca. 3 mm long. ***Column*** very short, ca. 1.5 mm long; stigma rounded, ca. 0.5 mm diam.; anther erect, smooth; pollinia not seen. ***Ovary*** green, glabrous, ca. 2 mm long. ***Capsule*** (immature) erect, ellipsoid or fusiform, 5–7 mm long, 2.5–3 mm diam. ***Seed*** not seen.

#### Phenology.

Flowering and fruiting observed in July.

#### Habitat and ecology.

**Figure 1.**
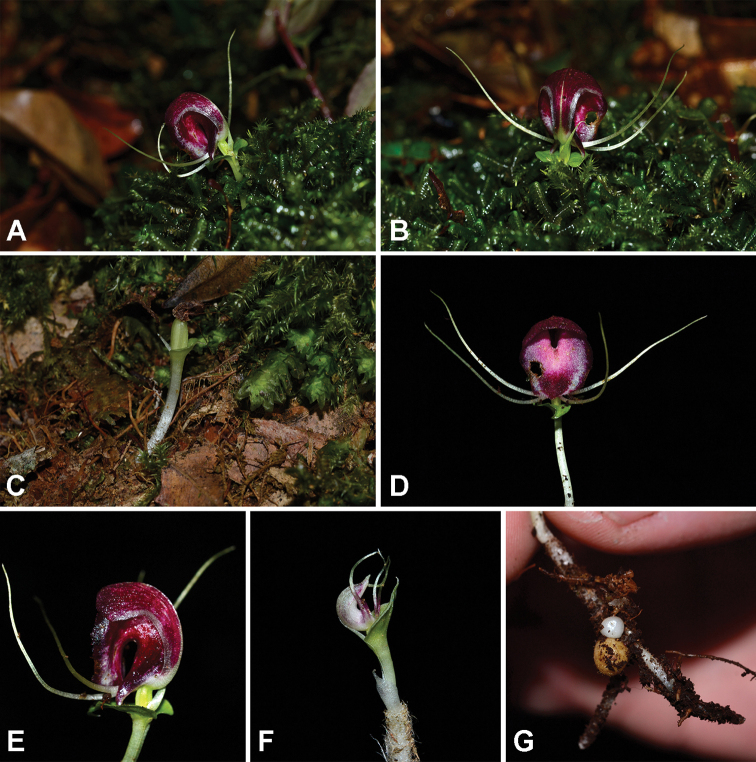
*Corybaspapillatus* Inuthai, Chantanaorr. & Suddee **A, B** plants in natural habitat, on humus associated with *Bazzania* sp. and *Acroporium* sp. **C** plant with immature fruit **D, E** flowers **D** front view **E** lateral view **F** immature flower **G** underground parts with tubers. Photographs by W. Kiewbang.

The new species was found growing amongst bryophytes (Fig. 1A–C), such as *Acroporium* sp., *Bazzania* sp., and *Schistochilaaligera* (Nees & Blume) J.B. Jack & Steph., on humus in shade in montane forest, ca. 1,700 m above sea level.

#### Distribution.

*Corybaspapillatus* is only known from the type locality (Fig. 3), however, it may also occur in other areas in peninsular Thailand with similar vegetation type.

#### Etymology.

The specific epithet ‘*papillatus*’ alludes to occurrence of irregular papillae in upper portion of abaxial (dorsal) surface of dorsal sepal.

#### Conservation status.

We consider it likely that if a formal assessment were performed, this species would be categorized as Critically Endangered (CR (D)) based on a preliminary risk of extinction assessment using the IUCN red list categories and criteria (IUCN 2019). This species is known from only four individuals from the type locality which attracts high numbers of camping tourists. Although we returned to the same locality and tried to find more specimens in 2020 it could not be found again. The species is, however, easily be overlooked in the field because of its small size.

**Figure 2.**
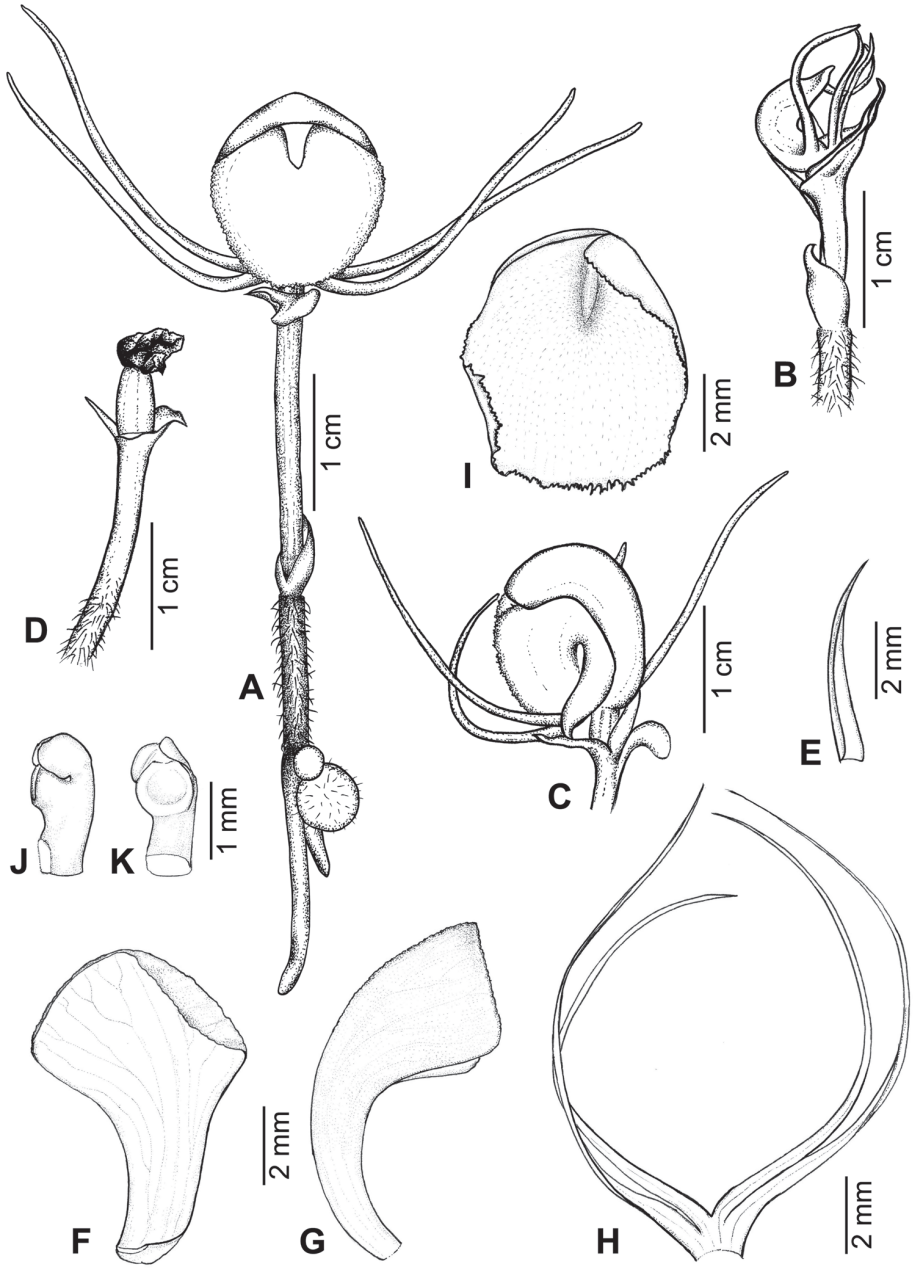
*Corybaspapillatus* Inuthai, Chantanaorr. & Suddee **A** whole plant with flower and tubers **B** plant with immature flower **C** mature flower, lateral view **D** plant with immature fruit **E** floral bract, **F, G** dorsal sepal **F** ventral view **G** lateral view **H** lateral sepals and petals **I** labellum **J, K** column, **J** lateral view, **K** front view. Drawn by J. Inuthai.

**Figure 3.**
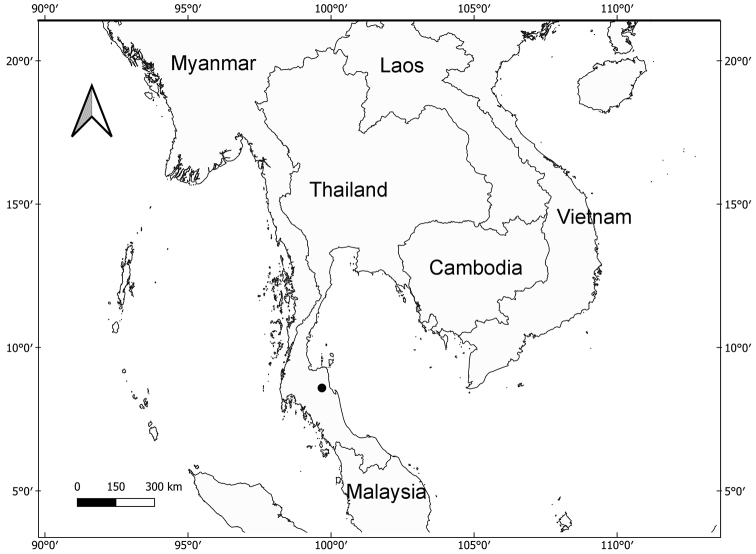
Type locality of *Corybaspapillatus* Inuthai, Chantanaorr. & Suddee (•).

## Discussion

*Corybaspapillatus* is most similar to *C.villosus*, which is endemic to Peninsular Malaysia (Dransfield et al. 1986; Go et al. 2015). These two species share several common features, *viz.* flower coloration being purplish, the dorsal surface of dorsal sepal covered with irregular papillae in the upper half, the hairiness of the labellum, the V-shaped throat, irregularly dentate to erose labellum margins, and well-developed conical spurs. *Corybaspapillatus* is distinguished from *C.villosus* by lacking dorsal sepal keel (strongly keeled in *C.villosus*) and the adnate lateral sepals and connate petals (all free in *C.villosus*).

*Corybaspapillatus* might be confused with *C.ridleyanus* Schltr., another endemic to Peninsular Malaysia (Dransfield et al. 1986; Go et al. 2015), which also has purplish flowers and reddish-purple labellum excepted for a zone below the margin, and a V-shaped throat. *Corybasridleyanus*, however, differs from *C.papillatus* by the truncate apex of the dorsal sepal and the free lateral sepals and petals.

Together with the recent discovery of a new species and new records of orchids from peninsular Thailand, especially in the Nakorn Si Thammarat mountain range (e.g. Ormerod et al. 2012; Tetsana et al. 2014; Chantanaorrapint and Chantanaorrapint 2016; Chantanaorrapint et al. 2017) it is clear that peninsular Thailand is an important region for orchid diversity and that further new species records can be expected to be found from many unexplored areas in this part of the country.

There are now three species of *Corybas* known from Thailand. A key to distinguish these is given below.

### Key to the species of *Corybas* in Thailand

**Table:** 

1	Lateral sepals and petals free at base; spurs inconspicuous, broad and poorly developed	***C.geminigibbus***
–	Lateral sepals adnate laterally to the connate petals at base; spurs well-developed, conical, slightly oblique to divergent	**2**
2	Flowers pink, dorsal sepal as long as the lateral sepals, acute at apex; central portion of labellum bearing a callus	***C.ecarinatus***
–	Flowers purplish, dorsal sepal shorter than the lateral sepals, rounded at apex; central portion of labellum hairy without a callus	***C.papillatus***

## Supplementary Material

XML Treatment for Corybas papillatus
